# Capillary Imbibition in Cementitious Materials: Effect of Salts and Exposure Condition

**DOI:** 10.3390/ma15041569

**Published:** 2022-02-19

**Authors:** Laurena De Brabandere, Natalia M. Alderete, Nele De Belie

**Affiliations:** Magnel-Vandepitte Laboratory, Department of Structural Engineering and Building Materials, Faculty of Engineering and Architecture, Ghent University, Technologiepark Zwijnaarde 60, Campus Ardoyen, B-9052 Gent, Belgium; laurena.debrabandere@ugent.be (L.D.B.); nataliamariel.alderete@ugent.be (N.M.A.)

**Keywords:** capillary imbibition, real-life exposure, porosity, concrete

## Abstract

Concrete structures are often exposed to harsh environmental conditions during their service life. Therefore, the investigation of transport properties and deterioration of concrete in different environments is an important topic. This paper reports the influence of salts (NaCl and Na_2_SO_4_) and exposure conditions (ideal laboratory (20 °C, 95% RH), a city and sea environment; including sheltered and exposed conditions) on capillary imbibition in cementitious materials with different water to cement ratios (0.4 and 0.6). First, the pore structure was assessed by water absorption under vacuum, torrent permeability, resistivity, and moisture content. The second part revolves around the capillary imbibition phenomenon with different imbibition liquids (water, NaCl, and Na_2_SO_4_). The results showed that, among the studied exposure conditions, sheltered conditions resulted in the largest porosity values and capillary imbibition rates (CIR). The influence of the imbibing liquid on the CIR depends on the w/c of the concrete. The CIR value for samples with a w/c of 0.4 is lower for Na_2_SO_4_ as imbibing liquid in comparison to water and NaCl. The sulfates might cause a pore blocking effect leading to a decreased CIR. For concrete with a w/c of 0.6, there was no significant difference between the different imbibition liquids. The influence of the pore blocking effect is probably smaller due to the larger porosity in this case. The findings of this research are important to understand the influence of real-life exposure conditions and therefore the influence of relative humidity, temperature, carbonation, and chloride ingress on the capillary imbibition phenomenon.

## 1. Introduction

Concrete structures are frequently exposed to harsh environmental conditions. For instance, a building along the coastline is exposed to salts that can penetrate into the concrete and cause deterioration [1]. In addition, a city environment can cause damage to reinforced concrete due to the high concentration of carbon dioxide (CO_2_) [2]. The ‘real life’ exposure conditions are in high contrast to the ‘ideal’ conditions often evaluated in the laboratory. The transport properties of concrete are important because deterioration is often caused by the ingress of aggressive substances (e.g., carbon dioxide, chlorides, and acids). Therefore, capillary imbibition is an important characteristic to assess the transport properties of concrete.

Test results for the rate of absorption of water can be influenced by several variables, such as the sample conditioning, the water to cement ratio (w/c), the paste volume fraction, and the use of partial cement replacements in concrete [3]. One of the largest impact factors is the initial moisture content of the sample (highly influenced by the environmental conditions). Castro et al. studied the influence of the relative humidity (RH) on the water absorption. During the sorption test, the pores of a sample conditioned at a lower RH can be filled more with water, since there is a greater volume of empty pores. Samples conditioned at 50% RH can have a ten times higher initial sorptivity (first 6 h of the test) than similar samples conditioned at 80% RH [3]. Porosity is another influential factor in the imbibition process. Benavente et al. (2018) described in their research the relationship between the porosity and capillary imbibition coefficient Cpar for different concrete mixtures. The obtained results indicate that higher porosity leads to a higher Cpar, which is due to a larger pore network, thus more water can be absorbed into the concrete [4]. Carbonation can also impact imbibition results due to the induced changes in the porosity. Carvajal et al. studied the relationship between accelerated carbonation, porosity, and capillary absorption in concrete made with pozzolanic Portland cement. Different time periods of accelerated carbonation and different w/c ratios were taken into account to study these relationships. The used exposure times for accelerated carbonation were 2, 4, 6, and 10 days. In this research, the results showed that an increasing carbonation time leads to an increasing absorption coefficient. This is probably caused by the formation of calcium bicarbonates during carbonation, that are more soluble in water and thereby increase the size of the capillary pores [5].

Although previous research on carbonation, porosity, compressive strength, and chloride ingress has been performed in on-site exposure conditions, there is no research that combines these factors and deterioration mechanisms with capillary imbibition. This means that in previous research on capillary imbibition, the environmental factors and deterioration mechanisms in the concrete were created in the laboratory. In the current research, the concrete samples were cured in a ‘real’ environment to provide better insight in the influence of the different factors combined. This was performed by means of indirect on site porosity measurements, measurements of carbonation depth, determination of chloride profiles, drying rate, and capillary imbibition experiments.

## 2. Materials and Methods

### 2.1. Materials

Two concrete mixtures were made with a different water to cement ratio (w/c): 0.4 and 0.6, denoted by PC4 and PC6, respectively. As the binder, Portland cement CEM I 52.5 N (Holcim, Brussels, Belgium) was added. The aggregates used were: sand 0/4, gravel 2/8, and gravel 8/16. The exact composition is shown in Table 1. To increase the workability of PC4, 2.9 L/m^3^ of superplasticizer (Masterglenium 51 con 35%, water reduction: ≥112% of reference mix and solid content of 35%, as indicated in the data sheet) was added. The mixing procedure consisted on mixing first the cement together with the fine and coarse aggregates for 1 min. Then the water was added and the mixing continued for 2 more minutes, finally the superplasticizer was added and mixed for 1 minute. From each mix, 22 beams at dimensions 400 mm × 100 mm × 100 mm and 30 cubes, with a 100-mm side, were cast. Immediately after, casting samples were placed in a curing room (20 ± 2 °C and 95 ± 5% relative humidity RH) and demoulded after 24 h.

The workability of both mixtures was tested using a slump test, as well as the air content and density. These fresh properties are shown in Table 2. For PC4 and PC6, the consistency class derived from the measured slump was respectively S2 and S4. The difference in slump between PC4 and PC6 can be explained by the different amount of water in the mix. In PC4, the superplasticizer was added to increase the workability of the mix while casting the samples. However, this did not lead to a slump as high as the one measured for PC6.

### 2.2. Curing Conditions

**Figure 1:**
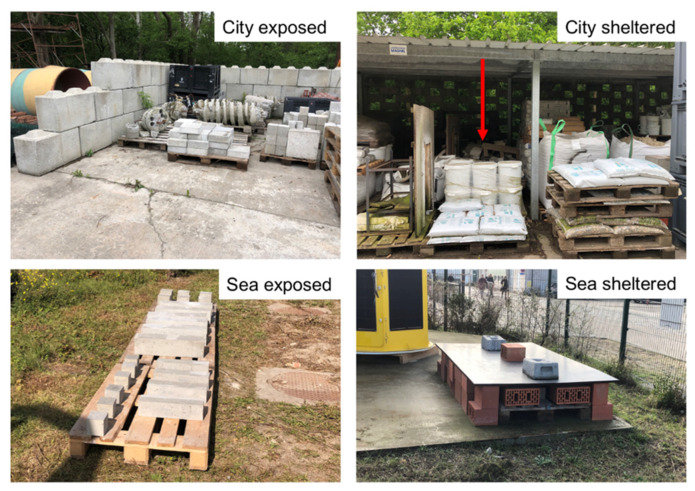
Curing and exposure conditions.

After demoulding, samples were cured and exposed in 5 different environmental conditions: ideal, city exposed, city sheltered, sea exposed, and sea sheltered (Figure 1).

In the case of ideal conditions, the samples were placed in the humid chamber of the laboratory with a constant temperature (20 ± 2 °C) and 95 ± 5% RH. The city sheltered and exposed samples were stored outside the Magnel–Vandepitte laboratory in Ghent. Samples in sea-sheltered and sea-exposed conditions were stored in Ostend, at the premises of VLIZ (Flanders Marine Institute), at a distance of 250 m from the sea. The sheltered samples were placed under a shed, whereby the conditions regarding temperature, RH, and CO_2_ concentration are the same in comparison to the exposed samples, however the samples were not exposed to rain and solar heat. For both mixtures, the exposure began in September. In each exposure condition, 8 beams and 12 cubes were placed: Half were tested after 2 months and the other half after 6 months of exposure. Such exposure times were chosen considering first keeping the samples on site for a long enough time in order to have an impact on the results before making the first measurements (2 months). A total of 6 months were chosen as the longest period of exposure because the hydration rate would then have become so low that further porosity alterations would be minimal.

### 2.3. Collection of Data from the Different Environments

At the coastline, several measuring stations perform continuous measurements on wind speed, temperature, RH, air pressure, etc. These data can be consulted on the Flemish Banks Monitoring Network [6]. Outside the laboratory, the temperature and relative humidity were measured using a data logger.

The data (Appendix A) showed that for an exposure time of 2 months as well as 6 months, the differences in temperature and RH between city and sea exposure were not significant. However, for the ideal environment, the average temperature and RH were significantly higher. These differences will have an impact on the curing of the concrete samples and thereby the porosity and compressive strength after 2 and 6 months exposure time.

### 2.4. Indirect Measurements of Pore Structure on Site

To describe the pore structure of the concrete cured in different environments, torrent permeability, resistivity, and moisture content were measured on the beams on site and on the beams in ideal conditions in the laboratory after 2 and 6 months of exposure.

1. The moisture content was measured using a non-destructive moisture content meter (TQC), which generates an electric field between 8 electrodes when pressed against the concrete surface. On each beam, 8 measurements were made;
2. Electrical resistivity was measured using the resipod (Proceq, Schwerzenbach, Switzerland). This non-destructive test is based on Wenner’s method and uses 4 electrodes with a spacing of 50 mm. On each beam, 4 measurements were made;
3. Torrent permeability was measured with the torrent permeameter (Materials Advanced Services Ltd., Buenos Aires, Argentina). A total of 8 measurements on each beam were made. This apparatus measures the air permeability in the concrete layer close to the surface. When some compaction defects were present at the surface of the beam, the measurement could not be carried out. This happened approximately 3 out of 50 times.

### 2.5. Capillary and Open Porosity

**Figure 2:**
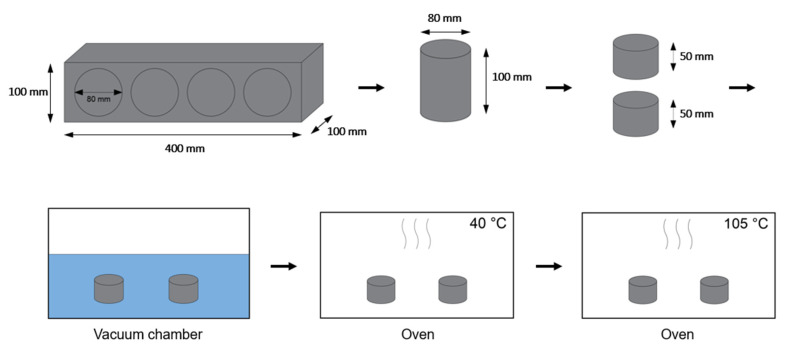
Schematic overview of the procedure (beam casting, samples cutting, immersion, and drying in oven) for water absorption under vacuum.

After the indirect porosity measurements on site, 4 cores with an 80-mm diameter were drilled out of every beam (height = 100 mm). For the determination of water absorption (WA), capillary porosity (CP), and open porosity (OP), the cores were cut in half, creating 2 cylinders with a height of 50 mm (Figure 2). The cylinders were put under vacuum for 2 h and then the vacuum chamber was filled with water until the samples were completely immersed. After 24 h of immersion, samples were taken out and weighed saturated in air (ma) and under water (mw). Finally, the samples were dried in an oven at 40 °C and weighed at regular intervals until the mass loss was lower than 0.1% in a 24-h period. The dry mass is designated as md40. Subsequently, the samples were dried at 105 °C. This dry mass is designated as md105 (*n* = 2 after 2 months exposure and *n* = 5 after 6 months exposure). WA, CP, and OP were calculated using the following equations:

(1) $$\mathrm{WA}\;\left(\%\right)=\frac{m_{a}{-m}_{d40}}{m_{d40}}\times 100$$

(2) $$\mathrm{CP}\;\left(\%\right)=\frac{m_{a}{-m}_{d40}}{m_{a}{-m}_{w}}\times 100$$

(3) $$\mathrm{OP}\;\left(\%\right)=\frac{m_{a}{-m}_{d105}}{m_{a}{-m}_{w}}\times 100$$

### 2.6. Carbonation Depth

**Figure 3:**
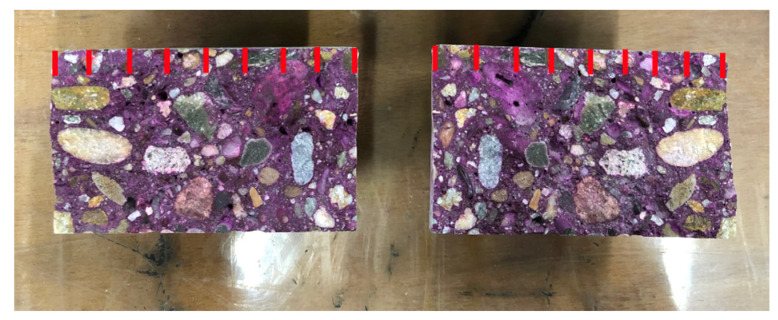
Measurements of carbonation depth on concrete samples.

For determination of the carbonation depth, 4 cylinders with a diameter of 80 mm and a height of 50 mm were split in half and subsequently sprayed with phenolphthalein. This indicator will turn purple at a pH higher than 9, which is the case for non-carbonated concrete due to the alkaline environment. On every sample, the carbonation depth was measured every 1 cm (Figure 3), resulting in 18 measurements for every exposure condition, time, and mix.

### 2.7. Determination of Chloride Ingress

The chloride content was determined in the samples that were exposed to the sea environment. The capillary imbibition test, explained in Section 2.9, was performed with water, chloride solution, and sulfate solution. The chloride content was also determined in samples cured in ideal conditions and exposed to the chloride solution during the capillary imbibition test. Three samples of sea-exposed conditions of PC4 and three more of PC6 were investigated after 2 months of exposure and again after 6 months. Three samples of sea sheltered conditions of PC4 and three more of PC6 were sampled after 6 months of exposure. In this case, no measurement was taken after 2 months because the chloride concentration in real conditions is much lower than for accelerated laboratory experiments, it was assumed that the chloride ingress would be limited at that time. The samples were used to grind several layers, creating a powder. The thickness of the layers depended on the exposure condition and exposure time. For the samples of PC6 after 2 months in a sea-exposed condition, 12 layers of 1 mm were ground. After performing the titrations, it was observed that chlorides did not reach a constant value in the deepest layers. Therefore the grinding was adapted for the remaining samples. The grinding pattern for every condition is shown in Table 3.

A potentiometric titration with silver nitrate (AgNO_3_) is used to determine the amount of chloride in the different layers. Thus, a chloride profile can be drawn that shows the amount of chloride in function of the depth from the exposed surface of the concrete. The titrations were done with the Metrohm 682 Compact Titrosampler, an automatic titration machine.

First, the powders were dried in an oven at 105 °C for at least one week. Then, around 2 g of powder was weighed and 5 mL of 0.3 mol/L nitric acid (HNO_3_) and 40 mL of demineralized water were added. The mixture was stirred and boiled before being filtered using a filter with a mesh size of 12–15 µm. After the whole mixture was filtered, demineralized water was added until the volume of the solution was 100 mL, the extract was shaken and stored. Then, 40 mL of 0.3 mol/L HNO_3_, 10 mL of demineralized water, and 10 mL of the prepared extract were combined in the plastic beakers of the titration machine. The titration equipment adds AgNO_3_, which reacts with the chloride ions in the solution and leads to precipitation of silver chloride (AgCl). When there is an excess of AgNO_3_, the titration is stopped.

### 2.8. Compressive Strength

Compressive strength was tested on cubic samples of 100 mm side. For each concrete mixture and exposure type, 3 cubes were tested after 2 and another 3 after 6 months of exposure.

### 2.9. Capillary Imbibition

For each exposure condition, cylinders with a diameter of 80 mm and height of 50 mm were used to test the capillary imbibition after 2 and 6 months of exposure (*n* = 12 after 2 months of exposure and *n* = 9 after 6 months of exposure). The lateral faces of the samples were covered with aluminum tape so the liquid can only enter the sample through the bottom surface during the capillary imbibition test described later in this section. Before starting the capillary imbibition test, the samples were preconditioned by immersion in water for 72 h. After immersion, samples were dried in an oven at 40 °C and weighed after 1, 2, 4, 6, and 24 h and then at regular intervals of 24 h. The drying was stopped when the weight change was below 0.1% over 24 h. From these measurements, the drying rate (DR) was determined as the slope of the linear regression line of the mass loss per exposed area (mg/mm^2^) as a function of the square root of time t^0.5^.

**Figure 4:**
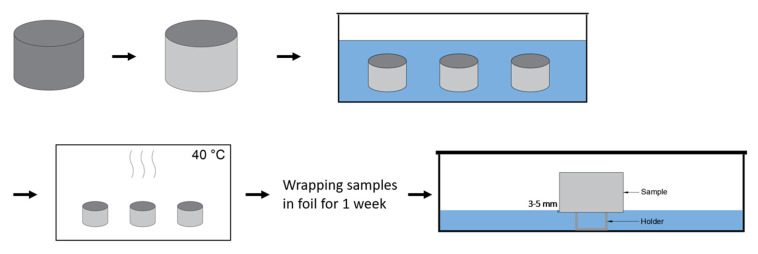
Schematic overview of the preconditioning (cutting, side-sealing, immersion, and drying) and the capillary imbibition test.

Then, samples were wrapped in plastic foil for a week to obtain a homogeneous distribution of moisture in the concrete. The imbibing liquids used in this research were water, a 3.5% NaCl solution, and 0.5% Na_2_SO_4_ solution. These concentrations were used because the average salinity in the North Sea is 3.5%. Sodium chloride covers approximately 78% of these salts [7]. As a result of this distribution, the concentration of the used Na_2_SO_4_ solution is smaller in comparison to the NaCl solution. The surface that had been exposed to the environmental conditions was put in contact with the solution. The samples were placed on holders and the level of the solution was kept 3–5 mm higher than the bottom of the sample (Figure 4). The samples were weighed after 0.5 h, 1 h, 2 h, 3 h, 4 h, 5 h, 6 h, and 24 h, and then every 24 h until the mass gain was lower than 0.1%. After this mass gain was reached, the samples were kept in the solution to see if there were any significant changes in the capillary imbibition. This resulted in a total of 10 weeks in the solution. Before weighing, the excess water was wiped from the bottom surface. The mass gain of the samples per exposed area (mg/mm^2^) was plotted as a function of the fourth root of time t^0.25^ (*n* = 4 after 2 months of exposure and *n* = 3 after 6 months of exposure) according to the new theoretical model Villagrán Zaccardi et al. (2017) [8] described in their research.

## 3. Results and Discussion

### 3.1. Indirect Porosity Measurements on Site

**Figure 5:**
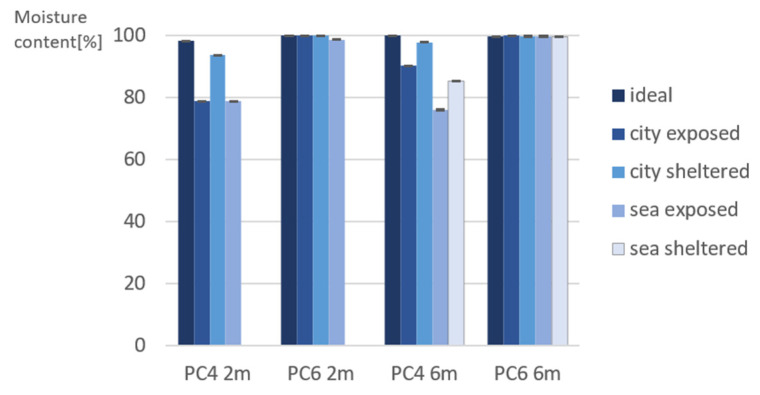
Moisture content measured on site (*n* = 16).

**Figure 6:**
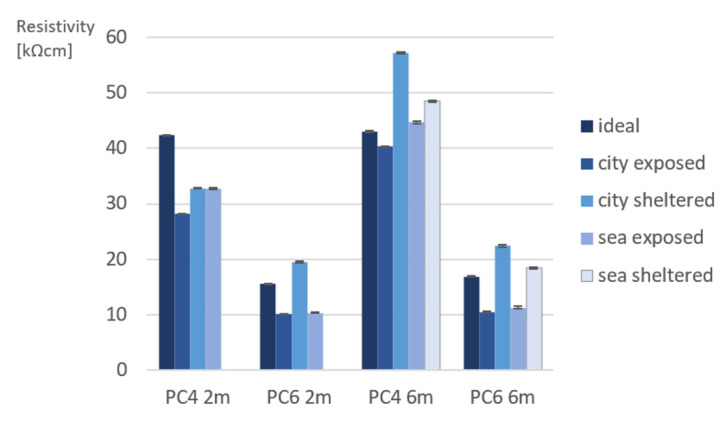
Resistivity measured on site (*n* = 8).

Figure 5 and Figure 6 shows the moisture content and resistivity measured on site after 2 and 6 months of exposure. Samples from PC4 2 and 6 months have a similar moisture content, however PC4 6 months has a higher resistivity than PC4 2 months. This can be related to the change in porosity of PC4 6 months in comparison to PC4 2 months. The slightly lower WA and OP values of PC4 6 months indicate a lower porosity and hence higher resistivity. For beams of PC6, the relative moisture content was 100% for almost every exposure condition and exposure time. This is because the porosity of these samples is higher due to the higher w/c ratio. Water and moisture can penetrate into the concrete more easily due to the larger pore network. When comparing the results of PC4, the moisture content was the lowest for beams in exposed conditions. This is most likely a result of the samples being more exposed to wind and warmth from the sun, which allows the moisture in the concrete layer close to the surface to evaporate more easily.

When comparing the resistivity of PC4 beams to PC6 beams, the resistivity measured of PC4 is significantly higher in comparison to PC6. This is because a lower porosity leads to a higher resistivity [9].

The results for torrent permeability measured on site are shown in Table 4. Torrent permeability for PC6 after 2 and 6 months of exposure was significantly lower for exposed conditions in comparison to sheltered and ideal laboratory conditions. This is because these measurements were made shortly after a period of rain, which caused the pores to be filled with water. Due to the water present in the pores, the air could not move through the pore network of the concrete and the value of air permeability was low.

Due to the different moisture content in the concrete beams, the measurements for moisture content, resistivity, and torrent permeability cannot be directly compared. In order to have relevant information on the pore structure, the beams should all have approximately the same moisture content. However, the obtained results give an indication of the amount of water that is present in the pore network, which is relevant for the interpretation of the results of the compressive strength test and carbonation depth.

### 3.2. Capillary and Open Porosity

**Figure 7:**
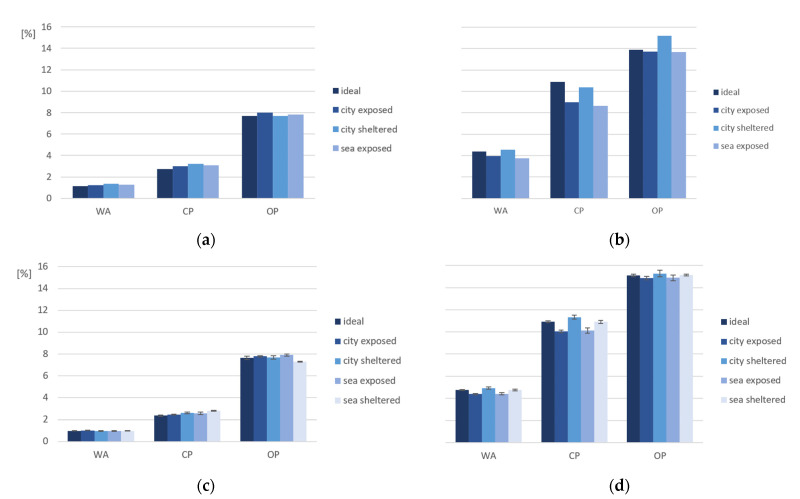
Water absorption (WA), capillary porosity (CP), and open porosity (OP) of (**a**) PC4 after 2 months (*n* = 2), (**b**) PC6 after 2 months (*n* = 2), (**c**) PC4 after 6 months (*n* = 5), and (**d**) PC6 after 6 months (*n* = 5). The standard deviation is represented by error bars.

Figure 7 shows the obtained capillary porosity (CP) and open porosity (OP) for PC4 and PC6 after 2 months and 6 months of exposure. After 2 months of exposure, the value for CP and OP is an average of 2 measurements. After 6 months, it was decided to test 5 samples to obtain a more accurate value.

As expected, CP and OP are higher for PC6 than PC4. This can be explained by the w/c of the concrete mixture. In concrete with a higher w/c, a part of the water in the mixture will not take place in the hydration reaction of cement and evaporate creating a larger pore network.

When comparing the results after 2 months of exposure, PC4 beams cured in ideal conditions have a slightly lower CP and OP. This is likely because there is more water available to continue the hydration reaction, which leads to a denser structure [10]. PC4 samples in city-sheltered conditions have the highest CP. The difference in CP and OP between the different exposure conditions is not significant. However, there is a slight difference. For samples of PC6, ideal samples have the highest CP after 2 months. Samples in city-sheltered conditions have the highest OP. CP in sheltered conditions is approximately 1.6% higher in comparison to exposed conditions, while the open porosity is approximately 1.5% higher. The difference in porosity between sheltered and exposed samples can be explained by the exposed samples often being exposed to rain. This provides water to continue the hydration reaction of cement, which causes a denser pore structure.

When comparing the results after 6 months of exposure, the difference in porosity of PC6 between the different exposure conditions becomes larger. Although it was expected that the ideal condition would more favorable for further hydration and the porosity of samples would be lower, this was not the case. The lowest porosity (capillary and open porosity) was reached for city-exposed and sea-exposed conditions. This is probably because the samples were exposed to rain, as previously mentioned. 

Regarding PC4, the same trend can be seen for PC6, however the differences are much smaller. Samples cured in ideal conditions have the lowest capillary porosity, which is due to the favorable RH and temperature. The capillary porosity is the highest for sea-sheltered samples. When looking at the open porosity (after drying at 105 °C), the lowest total porosity was calculated for sea-sheltered samples. The probable reason for the higher capillary porosity of the sheltered samples has already been explained with the results after 2 months of exposure.

Although it would be expected that porosity would decrease with time, it seems that for the case of a high w/c, porosity values are not so affected. It is surprising that some values of PC6 exposure conditions are even slightly higher. It is possible that the small differences are due to the limited amount of samples tested after 2 months, *n* = 2, in comparison to *n* = 6 after 6 months.

### 3.3. Compressive Strength

**Figure 8:**
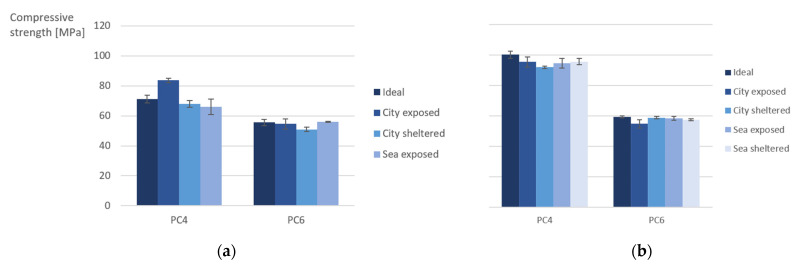
Compressive strength of PC4 and PC6 (**a**) after 2 months and (**b**) after 6 months of exposure (*n* = 3).

The compressive strength after 2 months (Figure 8a) is larger for PC4 in comparison to PC6 (level of significance 5.0%, *p* ≈ 98%), which can be explained by the lower porosity of PC4 due to the lower w/c ratio.

The highest compressive strength for PC4 is calculated for city-exposed conditions. This is unexpected and could be explained by the different moisture content in the samples when testing them. Chen et al. (2012) had similar results when studying the influence of moisture content on the compressive strength in concrete. In their research, the compressive strength decreased with an increasing moisture content. However, in concrete with a moisture content close to 100%, there was a slight increase of the compressive strength [11]. After 6 months of exposure (Figure 8b), there was a significant increase of compressive strength of concrete made with PC4. During curing, the hydration reaction of cement continued, which causes a higher strength. The highest compressive strength is measured for cubes cured in ideal conditions, with a value of 100 MPa and 59 MPa for, respectively, PC4 and PC6. After 6 months, the difference in strength (level of significance 5.0%, *p* ≈ 100%) between cubes made with PC4 and PC6 also increases with 110% in comparison to the difference after 2 months. Exposure time has a large influence on the compressive strength of samples made with mixture PC4. The statistical analysis shows that for PC6, there also is a significant difference (level of significance 5.0%, *p* ≈ 96%) between the compressive strength after 2 months and 6 months.

### 3.4. Carbonation Depth

**Figure 9:**
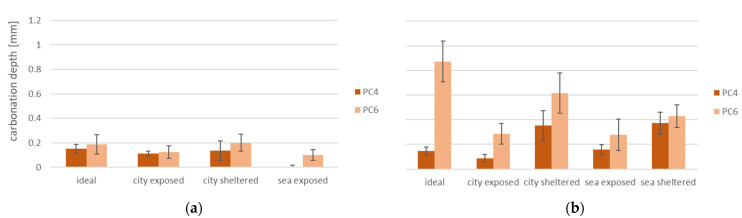
Carbonation depth of PC4 and PC6 (**a**) after 2 months and (**b**) after 6 months of exposure.

The results for carbonation depth after 2 months of exposure (Figure 9a) follow the statement that a higher porosity leads to a larger carbonation depth. This is because CO_2_ can penetrate more easily into concrete with a larger pore network. For both concrete mixtures, samples cured in exposed conditions have a lower carbonation depth than those cured in ideal or sheltered conditions. This is in line with research performed by Basheer et al. stating concrete surfaces exposed to rain have a lower carbonation rate [12].

A carbonation depth of PC6 grows significantly (level of significance 5.0%, *p* ≈ 100%) for every exposure condition after 6 months of exposure (Figure 9b) in comparison to results after 2 months. In samples made with PC4, there is a smaller increase (level of significance 5.0%, *p* ≈ 68%). For PC4 as well as PC6, the carbonation depth is smaller for samples in exposed conditions than for sheltered and ideal conditions. These samples are often exposed to rain, which blocks the pores, preventing CO_2_ to penetrate into the concrete. The progressing of the carbonation front generally creates a denser pore structure in the concrete. However, here the carbonation depth is limited to less than 1 mm, therefore the influence of carbonation on the compressive strength and transport properties will be limited. Since there were only two measurements of carbonation depth (after 2 and 6 months), it was not possible to calculate the carbonation coefficient. However, these results allow us to evaluate the influence of the different exposure conditions on the carbonation depth.

### 3.5. Chloride Ingress

**Figure 10:**
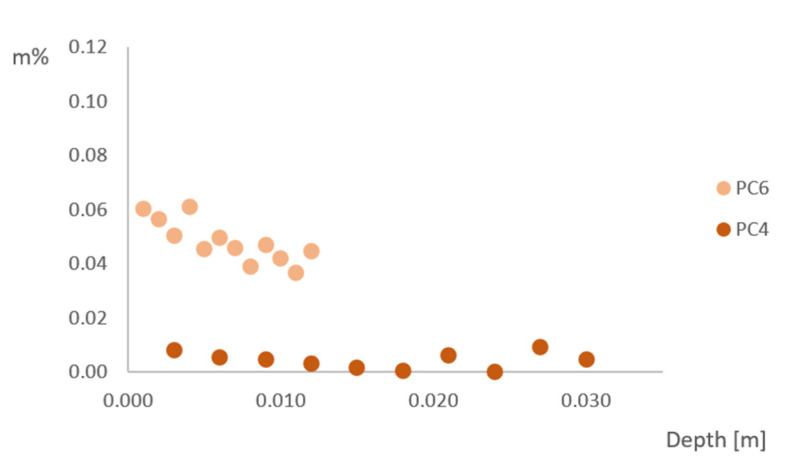
Chloride concentration as a function of depth for PC4 and PC6 sea exposed after 2 months of exposure.

**Figure 11:**
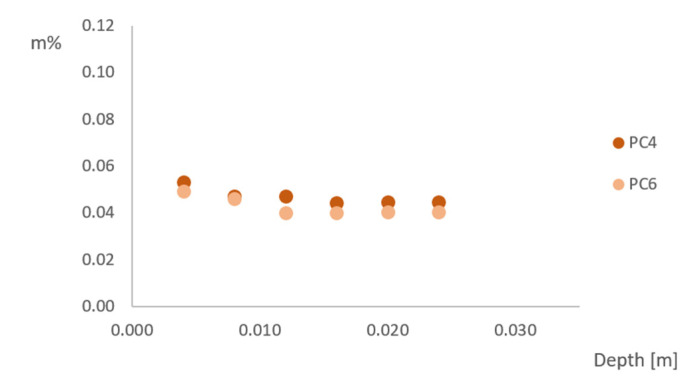
Chloride concentration as a function of depth for PC4 and PC6 sea sheltered after 6 months of exposure.

To determine the chloride ingress into the sample, titrations were performed for the samples in sea-exposed conditions after 2 and 6 months of exposure, for sea-sheltered conditions after 6 months of exposure and for the samples after capillary imbibition with NaCl. The amount of chloride (in m% of concrete) as a function of the depth (in m) in sea-exposed samples is shown in Figure 10 after 2 months of exposure. The chloride ingress in sea-sheltered samples after 6 months of exposure is shown in Figure 11.

**Figure 12:**
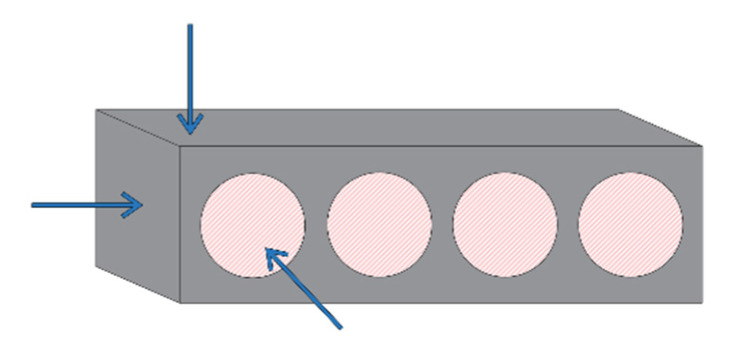
3D chloride ingress.

The chloride ingress in sea-exposed and sea-sheltered samples is due to diffusion. The chloride ions move from a region with a higher concentration (sea air) to a region of low concentration (concrete). The diffusion rate will be the fastest in saturated concrete, since diffusion of chlorides occurs through pore water present in the concrete [13]. When there is a one-dimensional chloride ingress, the graph can be described by Fick’s law. This would show a decreasing chloride content with increasing depth. However, this is not the case for the results of chloride ingress after 2 months for sea-exposed samples and after 6 months for sea-sheltered samples. The sea-exposed samples after 2 months show a slightly decreasing trend for PC6 and an approximately constant value for PC4. In this research, samples were exposed to three-dimensional chloride ingress. All sides of the beams were uncovered, leading to penetration of chloride ions in the concrete in all directions (Figure 12), which caused a more constant distribution of chlorides. Therefore, it is also not possible to calculate a diffusion coefficient. The chloride concentration in sea-exposed samples after 6 months exposure did not show an obvious trend as a function of the depth. For PC4, the chloride concentration had values between 0.00% and 0.06%. For PC6, the chloride concentration was in between 0.00% and 0.12%. This is probably an effect of the varying wet-dry cycles due to rainfall and dry periods (data in Appendix B). These cannot be described by Fick’s law. Note that results from PC4 and PC6 under sheltered conditions are similar, which is an unexpected result.

**Figure 13:**
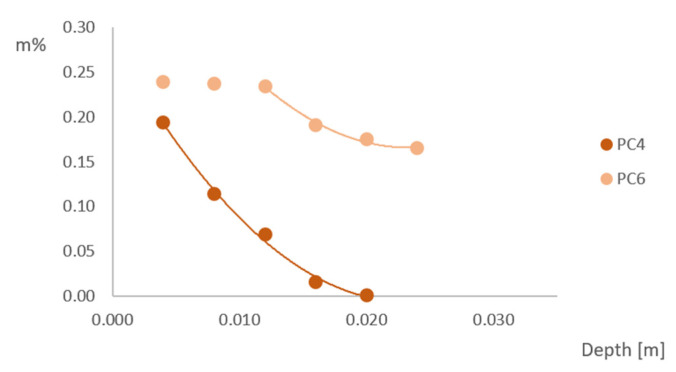
Chloride concentration as a function of depth after capillary imbibition with a 3.5% NaCl solution of PC4 and PC6.

As previously mentioned, the capillary imbibition test was also performed with a 3.5% NaCl solution with a duration of approximately 10 weeks and the chloride ingress in these samples was also tested. The chloride ingress in the samples occurs mainly through capillary absorption, not through diffusion (at least not as a dominant transport process at the beginning of the test). As shown in Figure 13, the chloride profile of PC4 samples seems to follow Fick’s law. For PC6 samples, the chloride concentration is a constant value in the layers close to the exposed surface. The chloride profile of the deeper layers also seems to follow Fick’s law.

Comparing the simulated chloride ingress in the laboratory (Figure 13) to the chloride ingress when exposed to a realistic sea environment (Figure 10 and Figure 11) shows that the on-site chloride ingress using beams of 100 mm × 100 mm × 400 mm is probably influenced by the 3D chloride ingress and dry and wet cycles and it cannot be compared to capillary absorption with a 3.5% NaCl solution.

### 3.6. Capillary Imbibition Rate (CIR)

**Figure 14:**
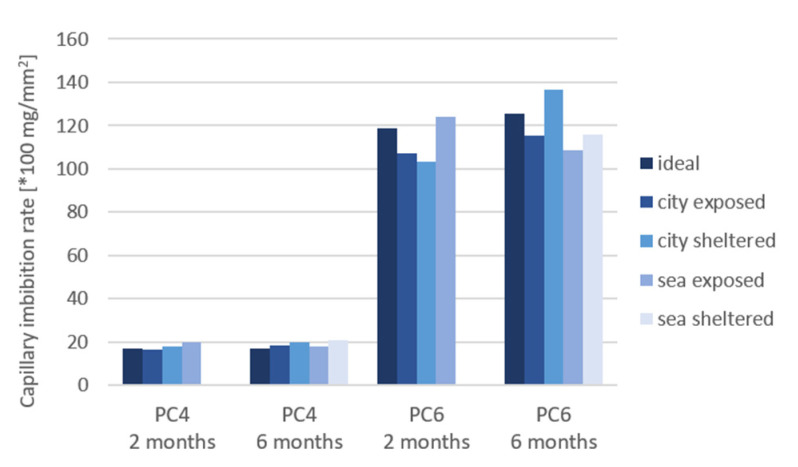
Capillary imbibition rate (CIR) of samples cured in different exposure conditions with water as the imbibing liquid.

**Figure 15:**
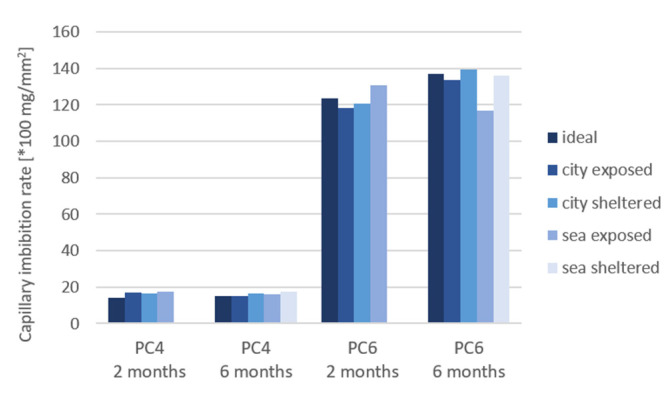
Capillary imbibition rate (CIR) of samples cured in different exposure conditions with a NaCl solution as the imbibing liquid.

**Figure 16:**
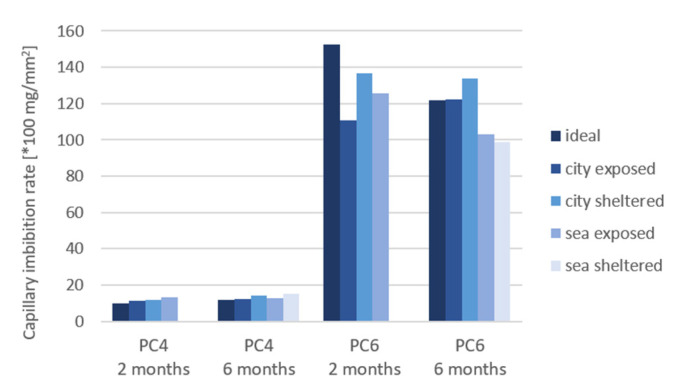
Capillary imbibition rate (CIR) of samples cured in different exposure conditions with a Na_2_SO_4_ solution as the imbibing liquid.

Figure 14, Figure 15 and Figure 16 shows the results for the capillary imbibition rate (CIR) of PC4 and PC6 samples with, respectively, water, a 3.5% NaCl solution and a 0.5% Na_2_SO_4_ solution as imbibing liquid after being cured in different exposure conditions for 2 and 6 months.

A statistical analysis was made to assess the significance of the results found in this research. An ANOVA test was used to assess the equality of means in different exposure conditions (Table 5). This test was made for every possible combination of imbibition liquid (water (W), NaCl (C), and Na_2_SO_4_ (S)), concrete mixture (PC4 (4) and PC6 (6)), and exposure time (2 months or 6 months).

The difference in CIR between the different exposure conditions is larger for PC6 in comparison to PC4. The difference becomes more significant after 6 months of exposure. The CIR is the highest for the sheltered conditions for all imbibition liquids, except for samples of PC6 in sea-sheltered conditions after 6 months of exposure in Na_2_SO_4_. This is probably because the exposed samples and samples in ideal conditions have more access to water to continue the hydration reaction. The same trend was found for the calculated porosity of the samples. For samples of PC4 with water as the imbibition liquid and 2 months of exposure, water as imbibition liquid and 6 months of exposure, and the Na_2_SO_4_ solution as the imbibition liquid after 6 months of exposure, the *p*-value is lower than or equal to 0.05. These values are underlined in Table 5. Which means that the mean values for the CIR of the different exposure conditions are statistically different. For these 3 situations, it could be statistically concluded that the CIR of samples in sheltered conditions is higher compared to the CIR in exposed or ideal conditions. The CIR did not reduce much from 2 to 6 months of exposure.

Comparing the different imbibing liquids, it can be concluded that the CIR is lower for the samples in the Na_2_SO_4_ solution than for water and the NaCl solution. It seems that the flow of water progresses more rapidly than the flow of the solutions. With the exception of the city-exposed condition, here, the CIR for water and NaCl are approximately the same. The slower uptake of Na_2_SO_4_ can be explained by a possible pore blocking effect and decrease of porosity that sulfates create when penetrating into the concrete [14].

An ANOVA test was also used to assess the difference between the 3 imbibition liquids (Table 6). For the samples of PC4 after 2 and 6 months of exposure, the *p*-values were 0.10%. These values are underlined in Table 6. This indicates a significant difference between the different imbibition liquids: water, NaCl, and Na_2_SO_4_. The CIR of tests performed with water is higher in comparison to NaCl, which is in turn higher than the CIR of tests performed with Na_2_SO_4_. This confirms the conclusions and possible explanations previously mentioned.

In case of both ANOVA tests, the significant *p*-values are for samples made with PC4. For PC6, the coefficients of variation (COV) are higher in comparison to PC4. This can explain why there is no significant difference found between the exposure conditions and imbibition liquids in statistical analysis.

## 4. Conclusions

1. Regarding the porosity evaluation of PC4 and PC6 under different exposure conditions, it was found that sea/city-exposed conditions lead to lower porosity values. This difference can be explained by the exposed samples often being exposed to rain. This provides water to continue the hydration reaction of cement, which causes a denser pore structure;
2. The carbonation depth is lower for samples cured in exposed conditions in comparison to sheltered and ideal conditions;
3. Chloride ingress in the tested samples does not follow Fick’s law (a lower chloride content deeper into the concrete), because of the three-dimensional chloride ingress in the rather small samples and the effect of wet-dry changes in real-life conditions;
4. The difference in the capillary imbibition rate (CIR) between the exposure conditions is more significant after 6 months in comparison to 2 months of exposure. After 6 months of exposure, the highest CIR is obtained for samples in sheltered conditions. The samples in ideal and exposed conditions have more access to water to continue the hydration reaction, leading to a lower porosity. Therefore the imbibition liquid cannot penetrate into the concrete easily;
5. CIR results of PC4 for Na_2_SO_4_ is lower in comparison to water or NaCl. This is probably because the sulfates create a pore blocking effect. The CIR of PC6 samples does not show this difference between the imbibition liquids, since the pores are larger, thus the decreasing pore size has a smaller effect.

The effect of on-site and laboratory-exposed conditions on concrete samples was evaluated through capillary imbibition. It seems that PC6 samples are much more influenced by the different environmental factors and that sheltered conditions are the least favorable for porosity development.

Further research to investigate the influence of exposure conditions on capillary imbibition should include increased exposure times to discern a more significant difference between the exposure conditions. In addition, microstructural changes or deterioration caused by real environmental conditions, such as carbonation and chloride ingress can be simulated in the lab under more controlled conditions to assess the influence on capillary imbibition.

## Figures and Tables

**Table 1:** Mix composition PC4 and PC6.

	PC4	PC6
Sand 0/4 [kg/m^3^]	673.8	616.4
Gravel 2/8 [kg/m^3^]	790.8	723.4
Gravel 8/16 [kg/m^3^]	561.3	513.5
CEM I 52.5 N [kg/m^3^]	325.9	325.9
Water [kg/m^3^]	130.4	195.5
Superplasticizer [L/m^3^]	2.9	-

**Table 2:** Fresh properties concrete.

	PC4	PC6
Slump [mm]	50	165
Air content [%]	1.7	1.7
Density [kg/m^3^]	2387.5	2331.25

**Table 3:** Grinding patterns.

Condition	Number of Layers (*n*)	Thickness Layers
Sea exposed PC4 2 months	12	1 mm
Sea exposed PC6 2 months	10	3 mm
Sea exposed PC4 6 months	10	3 mm
Sea exposed PC6 6 months	10	3 mm
Sea sheltered PC4 6 months	6	4 mm
Sea sheltered PC6 6 months	6	4 mm
Capillary imbibition PC4 2 months	6	4 mm
Capillary imbibition PC6 2 months	6	4 mm

**Table 4:** Torrent permeability measured on site (**a**) after 2 months of exposure (**b**) after 6 months of exposure (*n* = 16).

(a)			(b)		
Torrent Permeability [10^−6^ m^2^]			Torrent Permeability [10^−6^ m^2^]		
	PC4	PC6		PC4	PC6
Ideal	0.13 ± 0.03	25.43 ± 12.76	Ideal	0.15 ± 0.04	23.58 ± 10.78
City exposed	0.26 ± 0.05	0.48 ± 0.21	City exposed	0.19 ± 0.03	0.26 ± 0.09
City sheltered	0.15 ± 0.03	22.61 ± 11.45	City sheltered	0.21 ± 0.08	61.62 ± 14.73
Sea exposed	0.28 ± 0.06	0.13 ± 0.03	Sea exposed	0.78 ± 0.97	0.16 ± 0.04
			Sea sheltered	0.11 ± 0.02	13.41 ± 2.29

**Table 5:** ANOVA test for equality of means between different exposure conditions: *p*-value and average coefficient of variation (COV) (in %).

Situation	*p*	Mean COV (%)
W-4-2	0.05	5.20
C-4-2	0.19	5.80
S-4-2	0.15	13.15
W-6-2	0.11	10.49
C-6-2	0.074	13.10
S-6-2	0.47	21.27
W-4-6	0.005	4.45
C-4-6	0.346	7.20
S-4-6	0.017	7.29
W-6-6	0.45	15.14
C-6-6	0.55	12.22
S-6-6	0.42	19.83

**Table 6:** ANOVA test for equality of means between different imbibition liquids: mean value, *p*-value (in %) and COV (in %).

		µ (mg/mm^2^)	COV (%)	*p*
PC4 2 months	Water	0.161	7.59	0.10%
	NaCl	0.133	6.36	
	Na_2_SO_4_	0.076	6.54	
PC6 2 months	Water	1.186	9.55	24.30%
	NaCl	1.523	12.99	
	Na_2_SO_4_	1.436	28.67	
PC4 6 months	Water	0.171	5.17	0.10%
	NaCl	0.152	6.86	
	Na_2_SO_4_	0.117	6.73	
PC6 6 months	Water	1.253	16.04	74.20%
	NaCl	1.371	13.99	
	Na_2_SO_4_	1.218	26.91	

**Figure A1:**
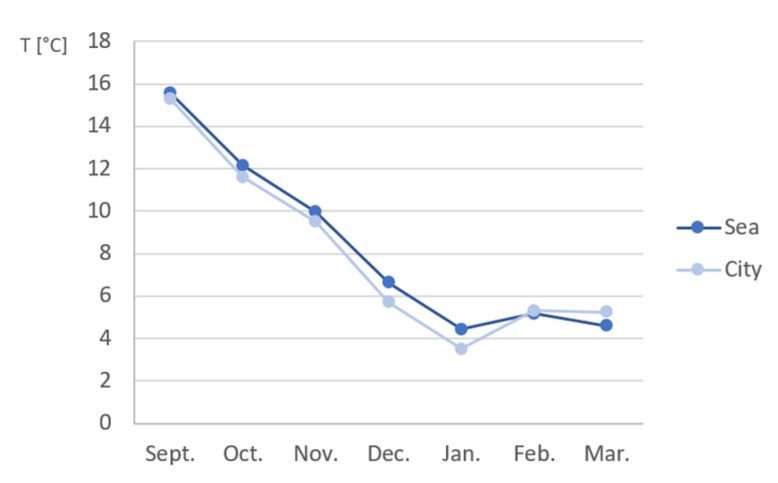
Average temperature in a sea and city environment during 6 months of exposure.

**Figure A2:**
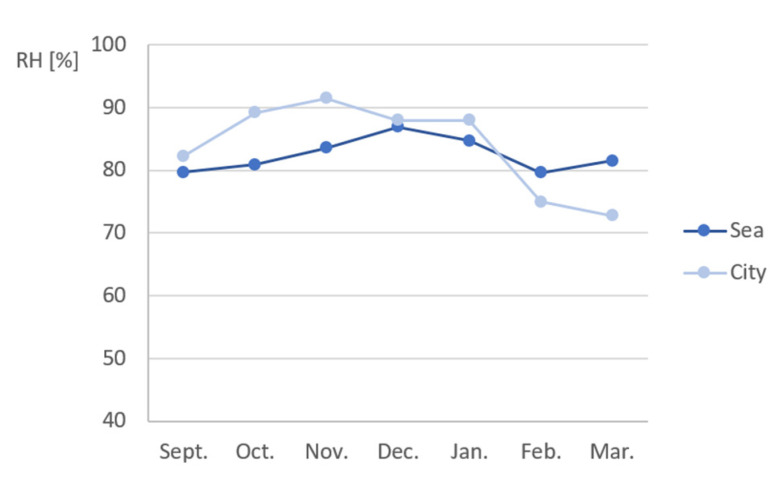
Average relative humidity in a sea and city environment during 6 months of exposure.

**Figure A3:**
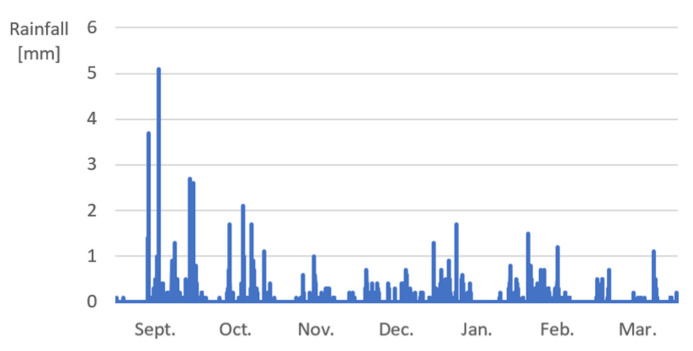
Rainfall during 6 months of exposure.

## Data Availability

Data will be made available upon reasonable request.
